# Estimation of Ebola’s spillover infection exposure in Sierra Leone based on sociodemographic and economic factors

**DOI:** 10.1371/journal.pone.0271886

**Published:** 2022-09-01

**Authors:** Sena Mursel, Nathaniel Alter, Lindsay Slavit, Anna Smith, Paolo Bocchini, Javier Buceta

**Affiliations:** 1 Department of Civil and Environmental Engineering, Lehigh University, Bethlehem, PA, United States of America; 2 Department of Industrial and System Engineering, Lehigh University, Bethlehem, PA, United States of America; 3 Department of Chemical and Biomolecular Engineering, Lehigh University, Bethlehem, PA, United States of America; 4 Department of Materials Science and Engineering, Lehigh University, Bethlehem, PA, United States of America; 5 Institute for Integrative Systems Biology (I2SysBio), CSIC-UV, Paterna, VA, Spain; Universita degli Studi di Trieste, ITALY

## Abstract

Zoonotic diseases spread through pathogens-infected animal carriers. In the case of Ebola Virus Disease (EVD), evidence supports that the main carriers are fruit bats and non-human primates. Further, EVD spread is a multi-factorial problem that depends on sociodemographic and economic (SDE) factors. Here we inquire into this phenomenon and aim at determining, quantitatively, the Ebola spillover infection exposure map and try to link it to SDE factors. To that end, we designed and conducted a survey in Sierra Leone and implement a pipeline to analyze data using regression and machine learning techniques. Our methodology is able (1) to identify the features that are best predictors of an individual’s tendency to partake in behaviors that can expose them to Ebola infection, (2) to develop a predictive model about the spillover risk statistics that can be calibrated for different regions and future times, and (3) to compute a spillover exposure map for Sierra Leone. Our results and conclusions are relevant to identify the regions in Sierra Leone at risk of EVD spillover and, consequently, to design and implement policies for an effective deployment of resources (e.g., drug supplies) and other preventative measures (e.g., educational campaigns).

## Introduction

Ebola Virus Disease (EVD), more commonly referred to as Ebola, is a hemorrhagic fever pathology that causes multiorganic failure followed by death (average fatality rate ∼50%) [1, 2]. EVD originates from a virus of the *Filoviridae* family discovered in 1976 after two consecutive outbreaks in Central Africa [3]. The accumulated evidence suggest that Ebola is a zoonotic disease with main reservoir hosts being fruit bats and non-human primates [4]. The first EVD outbreak is thought to have originated in a cotton factory and quickly transmitted to the relatives of first patients [5, 6]. The frequency of subsequent EVD outbreaks, approximately every other year since 1976, as well as their locations, overwhelmingly in the sub-Saharian region, reveals the dimension of a problem that is endemic to the African continent. New evidence hints at the possibility of latency as one of the mechanisms to explain this endemism [7]. As a matter of fact, at the time of preparation of this manuscript there were ongoing outbreaks in Guinea and in the Democratic Republic of Congo. Of all EVD outbreaks, the 2014–2016 one in West Africa was the most extensive and deadliest recorded ever [8]. The countries most intensely hit by the outbreak were Sierra Leone, Guinea, and Liberia; the case count of the West Africa outbreak was more than 27,000, with more than 11,000 deaths on record. This aggravated the conditions of communities already suffering from political instability, high rates of poverty, malnutrition, low life expectancy, and weak healthcare systems [9]. The outbreak spread also outside of Africa to Europe and the USA which increased the fear of a global pandemic and resulted in extensive public and media attention; the recent COVID-19 pandemic confirms that a global outbreak in our increasingly interconnected society is a serious and realistic threat. Indeed, the exponentially growing Ebola Virus epidemic in 2014 alarmed all the major health institutions, and on August 8^th^ 2014 the World Health Organization declared the EVD outbreak an international public health emergency [10]. As a result, health organizations, policy makers, and researchers were urged to understand and model the spread of Ebola in different contexts. Modeling efforts with a predictive character aimed at mitigating the effects of the epidemics have focused on Ebola virus pathogenicity from a molecular perspective [11, 12], the dynamics of the immune response [13, 14], human-to-human infection (including vaccination effects) [15–18], the effects of human mobility [19, 20], and also the ecological viewpoint [21–24].

Interestingly, there is abundant evidence that sociodemographic and economic (SDE) factors also affect, and can be used to infer, health and health-related behaviors, including disease propagation [25–27]. In that context, it has been shown that, typically, people with lower socioeconomic status have higher exposure to risk factors than the wealthier segments of the population [28]. While a consensus on the relationship between SDE factors and exposure to infectious diseases has not been reached [29], some modeling studies support the idea that poverty has an effect on the spread of infectious diseases [30–32]. However, we point out that this relationship is mostly supported by aggregate data at the country level (e.g., GDP) and not at the individual level. Still, a number of studies have explored the correlation between disease transmission and other indicators of the individual socio-economic status [33, 34]. In particular, Fallah et al. have shown in a study based on Liberia that individuals living in low income regions are more vulnerable to high rates of transmission and spread of Ebola [35]. Moreover, other studies concluded that the level of education is consistently associated with EVD epidemic size and spread [36], and that occupation is also correlated with the transmission of the Ebola virus [37].

Notably, only few studies have investigated the factors contributing to the likelihood of human beings exposed to Ebola virus from animal carriers. A recent study showed that the prominent behavioral factors associated with the transmission of the disease from animal to human (i.e., the infection spillover) are eating and/or hunting habits [38, 39]. This supports previous research that indicates direct contact with body fluids of Ebola infected animals is a substantial route of transmission [40]. More recently, some surveys led to an Ebola risk score based on perceptions and knowledge about the disease. In particular, Winters et al. measured the level of risk perception of survey respondents and aimed at shedding light on the relationship between risk awareness and the exposure to information sources [41]. Also, Wille and coworkers have recently analyzed the accuracy of assessing the zoonotic risk using virological data and they concluded that these analyses are incomplete, and that “surveillance at the human–animal interface may be more productive” [42].

**Fig 1:**
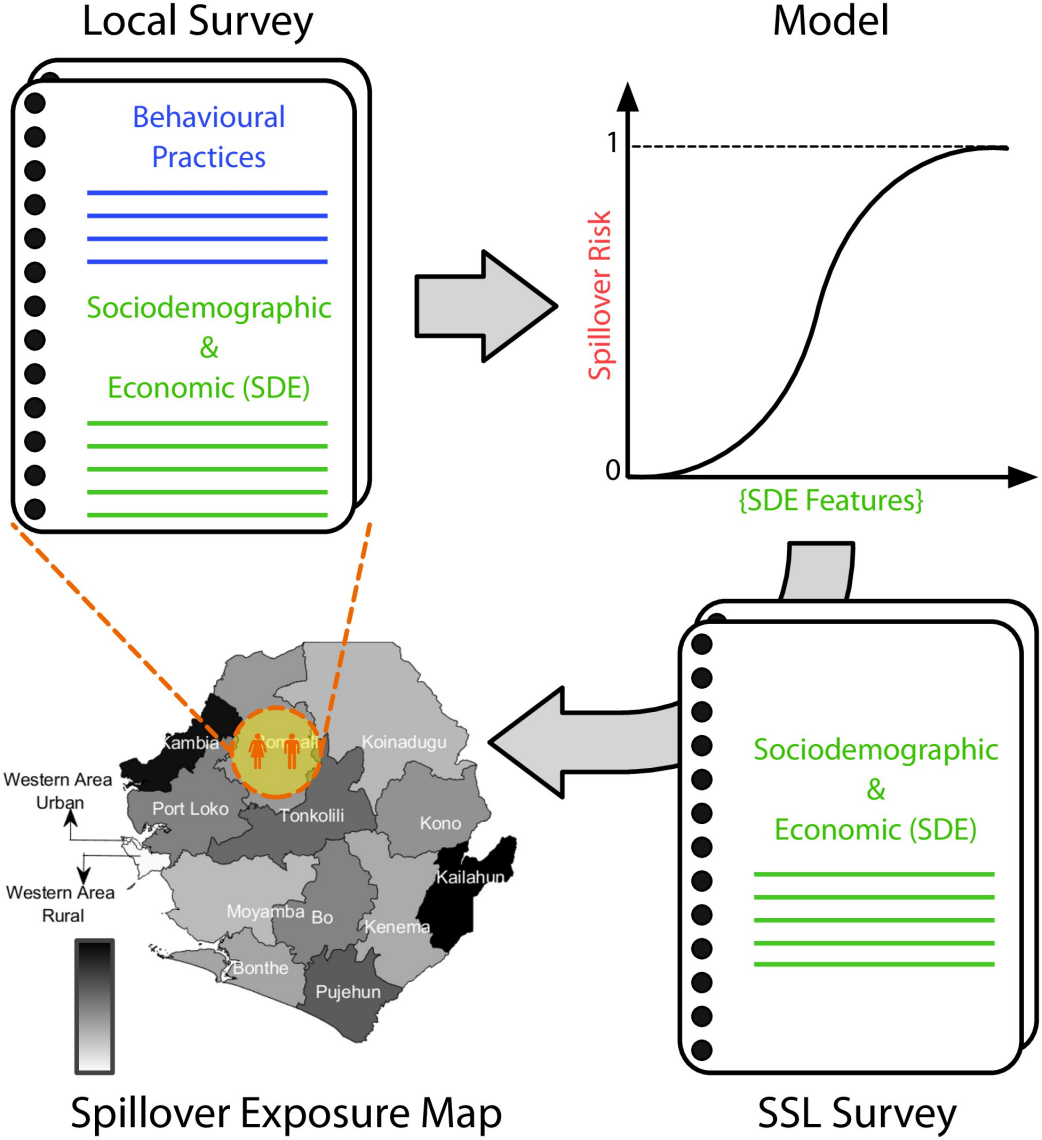
Methodological pipeline. We designed a survey that combines questions about behavioral practices that could expose individuals to Ebola infection and questions to measure sociodemographic and economic (SDE) factors. The survey was administered in Sierra Leone in the Bombali rural region. We analyzed our data by different means and developed a regression model that measures the spillover risk probability as a function of a number of SDE features. Once the model was calibrated, we extrapolated the results at the national level using surveyed data from Statistics Sierra Leone (SSL) to generate the infection spillover exposure map.

Altogether, previous works have identified determinants that increase the possibility of infection, but an association between the risky behavior of individuals and SDE factors has not been fully established. Herein we aim at bridging this gap of knowledge. To that end, we designed, collected, and analyzed survey data from one of the regions most affected by the 2014–2016 West African Ebola epidemic. By assessing simultaneously practices known to potentially cause animal-to-human transmission and socioeconomic/household traits, we define and measure, quantitatively, a spillover risk index. Since the individuals’ surveyed information is regularly measured by Statistics Sierra Leone (SSL) at the nation-wide level, our model, once calibrated, can be applied to other regions and times. Using this approach, we extrapolated the results to the entire country of Sierra Leone, see Fig 1. While, as reviewed above, the mechanisms driving EVD outbreaks are multifactorial, our methodology and results help to identify regions where spillovers are likely to occur. Thus, we expect our study to be relevant for EVD epidemic control, policy making, and planning of resource allocation (e.g., educational campaigns).

## Methods

### Geographical scope of the survey

**Fig 2:**
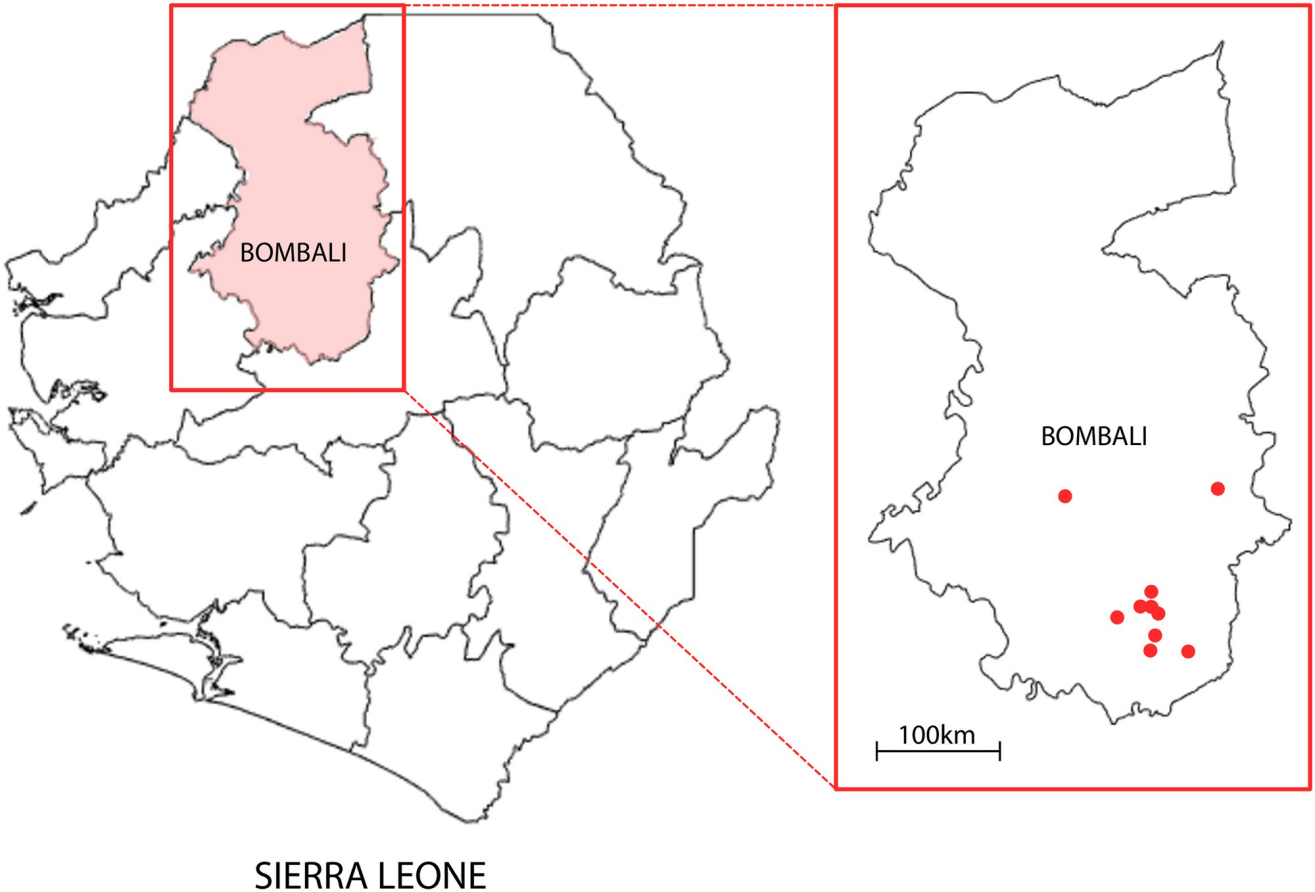
Survey locations in Sierra Leone. The survey was conducted in the district of Bombali over a period of three weeks. Ten different locations (red dots) were selected to obtain a representative sample of the population in rural areas of the country.

In the summer of 2019 we carried out a survey over 3 weeks in Sierra Leone. Sierra Leone was selected as the country of study as it is one of the countries most severely impacted by the 2014 Ebola epidemic [43]. The survey was conducted in collaboration with World Hope International (WHI), a NGO that aims at reducing poverty and improving health in Sierra Leone. The survey covered the district of Bombali, Fig 2. This district is located in the northwest region of Sierra Leone and was particularly affected by the 2014 Ebola epidemics [44, 45]. We focused on ten different locations (a city and several villages) that were suggested by WHI authorities due to their different levels of urbanicity, most common occupation, and other demographic characteristics of the residents. By doing this, we were able to obtain a diverse and representative sample of the population in rural areas of the country, which was our main target, due to their larger probability to have contact with wild-life, and hence increased probability of Ebola infection due to zoonotic sources. According to the last census conducted by Statistics Sierra Leone, the population of the district of Bombali is 606,544 (population density of 73/*km*^2^): 48.9% males, 71.5% of the population resides in a rural environment, 54.8% of the population is/was able to attend at least primary school, 63.5% of the population aged 15 years and over is economically active, and 11.7% of the population aged 10 years and over has access to the internet. The median age of the population is 18.7 [46]. As for our survey, the sample size was not defined a priori. As many adult individuals as possible were interviewed, given the time and resources available in Sierra Leone for the study. Thus, over the course of the 3 weeks, 284 respondents were surveyed. After excluding the first day respondents due to significant revisions to the survey questions (see below), 261 responses were utilized for the subsequent analyses. Guided by our local translators, we chose a random set of locations within driving distance from our operations center (Makeni). For each surveyed area, two teams went door-by-door following paths that were not predetermined. The response rate was extremely high and only a small number of individuals declined to take the survey. Our initial concern that surveying during working hours could skew the demographics of the respondents (by over-representing women and unemployed people) was quickly lifted, because we were able to show that the sample was representative of the demographics of the Bombali district and the rural areas of the country (see next section).

### Survey development and implementation

The survey instrument contained five different sections: *i*) sociodemographic characteristics, *ii*) household characteristics, *iii*) propensity of the respondents to behavioral practices leading to some risk of Ebola infection from an animal carrier, *iv*) environmental characteristics, and *v*) perception/knowledge of EVD. Sections *i*) and *ii*) measure SDE factors and were designed to match the data routinely collected by Statistics Sierra Leone (SSL) as part of their Demographic and Health survey which is conducted once every five years. Section *iii*) was developed based on current knowledge about the transmission of Ebola from animal carriers to humans. Section *iv*) assessed the presence of bats and other animal carriers in the surrounding environment. Finally, section *v*) measured each respondent’s perception and knowledge about Ebola.

In the United States, the survey was reviewed by a native from Sierra Leone, Mr. Vaafoulay Kanneh, to fine-tune the wording according to cultural practices and language differences. In addition, two scholars with extensive experience on the country and its culture reviewed the questionnaire and the survey administration strategy: Prof. Khanjan Mehta, Vice Provost for Creative Inquiry and Director of the Global Social Impact Fellowship program at Lehigh University, and Dr. Soumyadipta Acharya, Graduate Program Director of the Johns Hopkins Center for Bioengineering Innovation and Design, and Instructor of Biomedical Engineering at Johns Hopkins University. The survey was then reviewed by an independent scholar with experience in design and implementation of surveys, Dr. Jessecae Marsh, Cognitive Psychologist and Director of the Health, Medicine and Society program at Lehigh University, to ensure that the questions were effectively worded and not misleading.

Once on the ground in Sierra Leone, WHI provided 2 local translators to help with the administration of the survey. The translators were first surveyed as test subjects to confirm that the questions were clear from a Sierra Leonean perspective. They then translated the English version of the survey into Krio, the most commonly spoken language in Sierra Leone. The responses were then translated in their entirety to the team members from Lehigh University, who then transcribed the response for the questionnaire. The survey was administered in the form of face-to-face interviews: the translators would ask the questions to the respondent in Krio, who would respond in Krio, and the responses were transcribed in the questionnaire by the team members from Lehigh University.

Each survey took approximately 20–30 minutes to administer. Team members used the application *Fulcrum* [47] to record the responses, register the geographical location (GPS coordinates), and record the interviewee’s informed consent. Before each day of interviewing in the rural regions began, the two translators, as well as the team, would meet with the Chief of the village. This meeting was used to inform the leaders of the village of our presence and our purpose, as well as to get permission to conduct interviews in the village. In many cases, referencing this meeting encouraged respondents to take the survey and answer the questions more honestly.

Ethical permission for the survey (see S1 File) was granted by Lehigh University’s Institutional Review Board (IRB). The project received exempt status from the IRB, and both the survey and consent statement were submitted and approved prior to the trip and after the infield changes (see below). All survey participants were also offered paper copies of the informed consent in both English and Krio with the contact information of the principal investigators.

### On-site fine-tuning of the survey

The first day of surveying took place in the city of Makeni, very close to WHI’s local branch. We found that differences in African and Western cultures about the perception of “income” led to confusion. We also realized that our initial strategies to test the respondents’ knowledge about EVD were flawed. For instance, asking them to list potential mechanisms of EVD contagion often led to single answer responses and rarely to an elaborate list. Providing a list of actual transmission mechanisms and asking the interviewee to select if the option was correct or not led to many of respondents systematically accepting all options without thinking. To test each respondent’s knowledge more accurately, wrong answer choices were added to the survey’s final version. For example, in the final survey we added “witchcraft” as a possible answer choice when asking of a question about possible ways to get Ebola. The team also found that mentioning Ebola prior to asking questions about the disease resulted in some discomfort that might have affected the responses, presumably due to the stigma surrounding the disease throughout Western Africa. As a result of the first day of in-field experience, further changes needed to be made to the survey, and therefore the 27 interviews conducted that day are not used in future analyses. The questions regarding income were reworded to further reflect Sierra Leonean culture, the word Ebola was deliberately removed from the survey until it was specifically asked about. These changes resulted in the survey’s final form (Supplementary Material) which was administered starting the second day of surveying.

### Data preprocessing

Survey data was a combination of quantitative and qualitative (i.e., categorical) answers as a result of the nature of our questions (see S3 File). To incorporate all qualitative answers into our quantitative model (see Results), the former were associated with binary variables as follows. The answers to qualitative questions were grouped into categories. Then, one category, or one option in the multiple-choice questions, was chosen as the baseline. Each other option was associated with a binary variable (1 or 0). As a result, the number of variables used for each question was one fewer than the number of possible categories/options, to avoid redundancy. For example, under the work environment question, option ‘outdoors’ was chosen as the baseline and the other option (‘indoors’) was associated with a binary variable. So, for this specific question, value ‘1’ of the binary variable meant an ‘indoors’ occupation, and value ‘0’ meant an ‘outdoors’ occupation. Reference (i.e., baseline) categories/options were chosen to be either the one having largest number of responses (e.g, “Water from a well/pump” in the “ways for water acquisition” question), or the very first level of the answer options (e.g, “no formal education” in the “education level” question).

We set a threshold of 10 respondents for each possible answer category for each question to consider that category statistically significant. When this criterion was not satisfied, we merged answers into broader categories. For example, for the “water acquisition method” question, only four participants declared to purchase their water, so “Purchase” was put under the category “water_acquisition_other”. Similarly, all the other options with fewer than 10 responses were assigned to the “water_acquisition_other” category (see Table 1). For the question on the education level, as some choices had fewer than 10 responses (e.g., completed bachelors), but education levels are characterized by a clear rank, we regrouped the variables by similar levels. For example, “some primary school” had fewer than 10 responses and “completed primary school” had more than 10 responses, but, as they reveal a similar educational background, we grouped them in the same category. We used similar approaches while categorizing the other educational options and ended up with three categories (see Table 2).

**Table 1:** Water acquisition before and after data preprocessing.

Water Acquisition Ways	Assigned Categories
Purchase	water_acquisition_other
Running water in the house	water_acquisition_other
Water from a well/pump*	water_acquisition_water_from_a_well/pump
Water from a natural source	water_acquisition_water_from_a_natural_source

* Reference

**Table 2:** Education levels before and after preprocessing.

Education Levels	Assigned Categories
Arabic	education_primary
Completed Bachelors	education_high
Completed Diploma or Postsecondary Training	education_high
Completed Junior Secondary School (JSS)	education_secondary
Completed Masters or Doctorate	education_high
Certificate	education_high
Completed Primary School	education_primary
Completed Senior Secondary School (SSS)	education_secondary
Mason	education_primary
No Formal Education*	education_no_formal_education
Some primary school	education_primary
Trade school	education_primary

* Reference

For the question aiming to know the respondent’s occupation, the answers were spread over 22 different options, which did not reveal a clear grouping by sector. Since their combination would lead to too broad categories, which could harm the predictive capability of our model, these specific answers were ignored, and only the answers to the indoors/outdoors question (Question A7) were used to describe the occupation. For the question asking the district of birth, 84% of the responses were “Bombali”, as expected. Thus, the significance of the question was deemed minimal and so we did not include it in our analysis.

Some questions implied time frequencies, such as the question about the average internet usage. In this case, the responses were converted into numerical values (between 0 and 1) that describe the number of occurrences per day, e.g. “at least once a week” was converted to 1/7 (see Table 3). Numerical variables (e.g., age) were divided by their corresponding maximum values to make them dimensionless.

**Table 3:** Internet use before and after preprocessing.

Internet Use	Assigned Categories
At least once a day	1
At least once a week	1/7
At least once a month	1/30
Less than once a month	1/60*
Not at all	0

* This was set as the average of the values in answers “At least once a month” and “Not at all”.

Finally, regarding location information, the GPS coordinates are available in Fulcrum records. We noticed large variability in the “average time to highway” responses, and measuring the distance (in miles) from households to the nearest highway indicated the responses to this question were generally inaccurate. In particular, we expected that the average time to highway from similar locations (i.e., same villages) to be similar and we compared the responses with our distance measurements. We found that the coefficient of variation of the “average time to highway” responses located in same villages was larger than 1 in most cases. Hence, we omitted this variable (“average time to highway”) from the final data set that we used in our analyses.

In summary, taking into account the references/baselines, the final data set included 1 option for gender, 3 options for the education level, 1 variable for religion, 1 variable for work environment, 2 variables for relative income, 2 variables for water acquisition ways, 2 variables for ownership of cell phone as binary variables; and included the frequency of internet usage, age, the number of rooms in house, the number of people in household, average time to school, average time for fuel, and average time for water as numerical variables (See S4 Data).

### Evaluation of the reliability of the data

Our collected data shares sociodemographic and economic information with one of the surveys regularly performed by SSL (Sierra Leone Integrated Household Survey: SLIHS) [48]. On the one hand, this allowed us to check if our survey was representative to capture the sociodemographic statistical data of the Bombali district where we ran our survey, and also of Sierra Leone in rural areas. On the other hand, as shown below, this provides the means to extrapolate the applicability of our quantitative regression model to the whole country.

**Fig 3:**
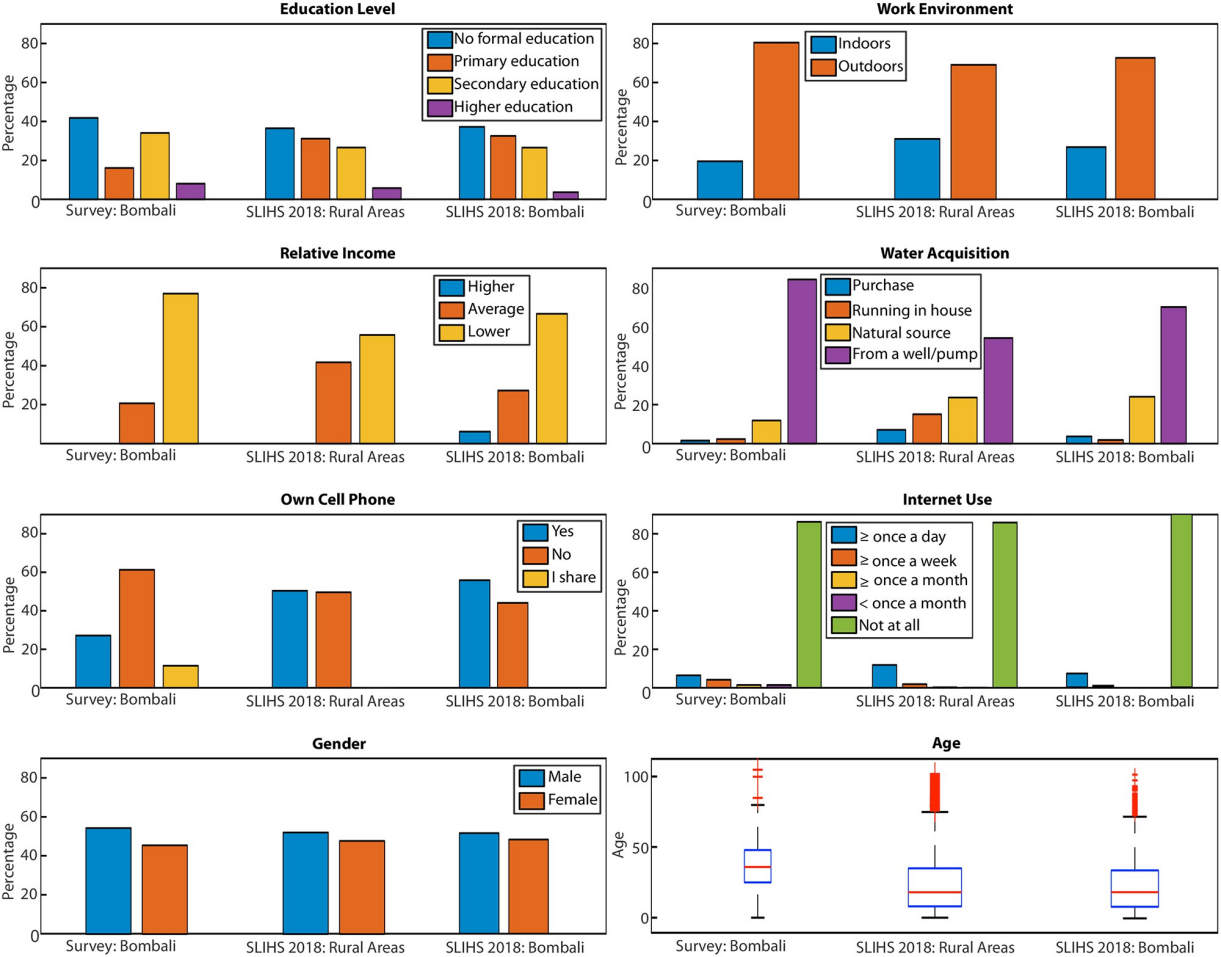
Comparison of the distributions in rural areas between our survey (Bombali district), SLIHS 2018 in rural areas at the country level, and SLIHS 2018 in the Bombali district. From top to bottom and from left to right: education level, relative income, cell phone ownership, gender, work environment, water acquisition method, internet use, and boxplot of age (median: central red line; bottom and top box edges: 25^th^ and 75^th^ percentiles, respectively; outliers: plus symbols).

For this comparison, we used the 6 features (variables) that were deemed as representative in our regression analysis (see Results) as well as “Gender” and “Age” (Fig 3) because they are prominent demographic characteristics. Also, to compare our survey with the SLIHS 2018 at the country level, we filtered out from the data from the Western Area Urban district (i.e., the capital Freetown). In fact, including such data would consider individuals with SDE characteristics that differ significantly when compared to rural areas, which are the focus of our research. Fig 3 shows that the overall trends of the demographic features are qualitatively matched. For a quantitative comparison of continuous variables (e.g., age), the Kolmogorov-Smirnov (KS) test was used to examine the similarity of the distributions from our survey and from the Bombali district data, as well as the whole nation. The KS index measures the degree of uniform converges of the two distribution functions, so it is a particularly difficult discrepancy metric to minimize. The results indicated that the age distribution was captured very well both in the Bombali district and entire nation, with a maximum discrepancy of 4.8% and 4.5%, respectively. Time to school was captured sufficiently well both at the district and country level, with maximum discrepancies of 18% and 15%, respectively. The number of rooms in the house was also represented sufficiently well, with KS statistics of 25% and 35%. The time required to collect water was not captured well (maximum discrepancy 54% and 56%), but we had noticed that this metric was affected by a large degree of subjectivity in its assessment (answers varied significantly among respondents residing in the same area). For this reason, we judged it as unreliable and we did not use it in our final model. The categorical variables (e.g., gender, work environment, religion, relative income) were tested for similarity by computing the dissimilarity index (i.e., total variation distance), the distributions overlap, the Bhattacharyya coefficient, and the Hellinger’s distance. Alike distributions yield values of the dissimilarity index and the Hellinger’s distance close to zero and values of the distributions overlap and the Bhattacharyya coefficient close to 1. The results for the distributions of the most important categorical variables are given in Table 4. Table 4 shows similarity of the distributions coming from our survey and the SSL survey both for the district of Bombali, and the whole country. Overall, the analyses suggest that our survey was representative of the Bombali district demographics and, more importantly, that our extrapolation to capture the spillover risk at the national level is meaningful (with the exception of the Western Area Urban district that we excluded from our analysis).

**Table 4:** Results of marginal distributions of categorical variables.

Variable	Dissimilarity Index (range 0–1, lower is better)		Overlap between distributions (range 0–1, higher is better)		Bhattacharyya coefficient (range 0–1, higher is better)		Hellinger’s distance (range 0–1, lower is better)	
	Bombali	Country	Bombali	Country	Bombali	Country	Bombali	Country
Gender	0.028	0.022	0.97	0.98	0.99	0.99	0.019	0.016
Religion	0.25	0.23	0.75	0.77	0.96	0.97	0.20	0.18
Work environment	0.38	0.43	0.62	0.56	0.92	0.89	0.29	0.32
Relative income	0.10	0.21	0.90	0.79	0.99	0.97	0.093	0.16
Education	0.16	0.15	0.84	0.85	0.98	0.98	0.15	0.13

### Risk index assessment

An important quantitative output of our survey was the Ebola spillover risk index, *RI*, a number that measures the likelihood of an individual to engage in behaviors that can lead to contracting Ebola virus from an animal host. The risk index was calculated for each individual respondent using nine questions from the section specifically related to these behaviors and five questions from the Ebola perception section. The contributions to the risk index resulting from these questions were assessed in different ways (see below) and provided the partial indexes *RI*_1_ and *RI*_2_ that were combined to obtain the value of *RI* for each respondent. Table 5 collects the questions that were used to estimate *RI*_1_ and the scores $r_{i_{1}}$ associated with each of the possible answers: $RI_{1}=\sum r_{i_{1}}$. As shown in the table, the score for each question lies within the [−1, 1] range.

**Table 5:** Risk scores $r_{i_{1}}$.

Question #	Question	Answer	$r_{i_{1}}$
C2b*	How often do you go to caves?	Never	-1.0
		Every few years	-1.0
		Every few months	0.0
		Every few weeks	1.0
		Every few days	1.0
		Every day/more than once per day	1.0
		Declined to answer	0.0
C3	How often do you wash with soap?	At least once a day	-1.0
		At least once a week	0.0
		At least once a month	0.0
		Less than a month	1.0
		Never	1.0
C5	When you eat fruit, do you check if it has been bitten by animals?	Always	-1.0
		Sometimes	0.0
		Never	1.0
		I don’t eat fruit	0.0
C9	How often do you eat bushmeat?	Every Meal	1.0
		Once a day	1.0
		At least once a week	1.0
		At least once a month	1.0
		At least once per year	0.5
		Never	-1.0
		I used to, but no longer	-1.0
C11	Do you clean your hands before eating?	Always	-1.0
		Sometimes	0.0
		Never	1.0
C13	How often do you spend time in places where bats nest?	Never	-1.0
		Every few years	0.0
		Every few months	0.5
		Every few weeks	1.0
		Every few days	1.0
		Every day/more than once per day	1.0
C14	How often do you have contact with someone else’s blood or bodily fluids?	At least once a day	1.0
		At least once a week	1.0
		At least once a month	0.0
		Less than a month	0.0
		Never	-1.0
C15	Do you believe that touching raw meat or any live animal could spread disease?	Yes	-1.0
		No	1.0
		I don’t know	0.5
C16	Do you believe that eating bushmeat could spread disease?	Yes	-1.0
		No	1.0
		I don’t know	0.0
E2	Do you think a person could get Ebola from an animal, dead or alive?	Yes	-1.0
		No	1.0
		I don’t know	0.5
E7	Do you believe that you can get Ebola from bushmeat?	Yes	-1.0
		No	1
		I don’t know	0.5

* This question was asked only to the participants who answered “Yes” to question C2, “Do you know any caves?”.

Specifically, every answer gets a score of −1, −0.5, 0, 0.5 or 1 depending on the level of exposure to infection. If an action reveals a risky behavior, we assigned a score of 1, and if the behavior decreases the likelihood of infection, then −1 was assigned. For questions where answers imply a time frequency (e.g., “every day”), the score of the riskiest answer was given 1 and the score of −1 was assigned to the least risky answer (intermediate answers were given one of the other 3 possible values mentioned above). The second contribution to the risk index, $RI_{2}=\sum r_{i_{2}}$, was determined based on “check all that apply” type of questions (questions E1, E2b, and E3 of the survey, Table 6). The possible options for these three questions included both correct and wrong answers on mechanisms of human-to-human Ebola infection, animal-to-human Ebola infection, and strategies to prevent Ebola. As mentioned above, wrong answers were included in these questions after we evaluated the conducted interviews of the first day and we noticed that a number of respondents checked all choices. We then modified the questions by providing multiple options that included both correct and wrong answers. Using the modified survey, the scores $r_{i_{2}}$ were assigned using the following procedure.

1. If a respondent gave more than one wrong answer to a question, then $r_{i_{2}}=1$.
2. If a respondent gave only one wrong answer to a question and could not provide at least half of the reasonable answers, then $r_{i_{2}}=1$.
3. If a respondent gave only one wrong answer to a question but provided at least half of the reasonable answers, then $r_{i_{2}}=0.5$.
4. If a respondent gave only correct answers, then $r_{i_{2}}=-1$.
5. If a respondent answered “I don’t know”, then $r_{i_{2}}=0.5$.

**Table 6:** Risk scores $r_{i_{2}}$.

Question #	Question	Answer
E1	What are the ways in which a person gets Ebola?(Check all that apply) (Open Question)	By air
		Bad odor or smell
		Preparing bushmeat as a meal
		Eating bushmeat
		Eating fruits likely to have bitten by bats
		Eating with an infected person
		The saliva of an infected person
		Blood of an infected person
		The sweat of an infected person
		The urine of an infected person
		Feces of an infected person
		Living with an infected person
		Working with an infected person
		God’s will
		Witchcraft
		Government hoax
		Ebola does not exist
		I do not know
		Declined to answer
E2b	How could a person get Ebola from an animal? (Check all that apply) (Read options)	Having an animal as a pet
		Eating any meat
		Eating bushmeat
		Watching an animal
		Eating fruits bitten by an animal
		Hunting
		Preparing bushmeat as a meal
E3	In general, how do you think a person avoids Ebola? (Check all that apply) (Read Options)	Brushing their teeth
		Sleeping under a mosquito net
		Avoiding contact with blood and bodily fluids
		Drinking tea
		Staying inside when it rains
		Not touching anyone with the disease
		Cleaning themselves with soap and water
		Avoiding funerals or burial rituals
		Drinking only tap water
		Avoiding the forest/woods
		I don’t know
		Declined to answer

### Regression analysis and machine learning techniques

One goal of our study was to develop a methodology able to determine the risk index *R* not just for individuals that took our survey, but also for individuals for which SDE information is part of the publicly available data from Statistics Sierra Leone (SSL). To that end, we calibrated a model that takes as *input* the answers to the same SDE questions from the survey of SSL and returns as *output* the risk index, *RI*. We calibrated and tested multiple models via regression analysis and supervised machine learning, in which the risk index was used as a response variable for training and the other answers were used as features. For the regression analysis, we tested a multiple linear model, a second order multivariate polynomial model, and a logistic model. In linear and polynomial regression, the output of the models was chosen to be the risk index, *RI*. On the other hand, when using the logistic regression approach and machine learning classification techniques, the model was not trained using the actual value of the *RI*, but a binarized description (*RI*_*b*_) of the continuous risk index by classifying the respondents into “high risk of spillover exposure” and “low risk of spillover exposure” (based on whether *RI* was above or below the average risk index). In this way, we simplified the output of the predictive algorithm, settling for a classification of high or low risk, rather than a full quantification of *RI*.

#### Multiple linear regression model

In this case, a full regression model would read,

$$RI=\beta_{0}+\beta_{1}X_{1}+\beta_{2}X_{2}+\cdots +\beta_{n}X_{n}$$

where RI is the spillover risk index, and *X*_1_, *X*_2_, …, *X*_*n*_ are the predictor variables. Since the data set includes 19 sociodemographic features, a full regression analysis would result in 20 parameters to be calibrated (i.e., *β*_0_, *β*_1_, …, *β*_19_). However, we deemed this amount to be too large for an effective calibration, considering that the total number of observations was 261, and this was confirmed by the the adjusted *R*^2^ values. In order to overcome this problem, we implemented a dimensionality reduction approach (feature selection) by exhaustive search, forward/backward stepwise, and sequential replacement (*regsubsets* function of the R package ‘*leaps*’) [49]. This provided the best subsets of the variables in the dataset in terms of predicting capabilities for the continuous *RI*. The best model was found to have only 6 variables. In particular, the model performance was evaluated by computing the adjusted *R*^2^ value, but even for the best model we obtained a value of 0.073, which was clearly too low.

#### Second order multivariate polynomial regression model

In this model, the linear behavior is extended with second order interactions among predictor variables,

$$\begin{array}{c} RI=\beta_{0}+\beta_{1}X_{1}+\beta_{2}X_{2}+\beta_{3}X_{1}^{2}+\beta_{4}X_{2}^{2}+\beta_{5}X_{1}X_{2}+\ldots \end{array}$$

Regardless of its simplicity, this kind of models have been shown to capture complex interactions satisfactorily, such as those of enviroclimatic features to determine the carrying capacity of bat species [24]. We used the same approach described for the linear regression model and performed a dimensionality reduction to investigate the best subset of predictors (*regsubsets* function of the R package ‘*leaps*’). The best model in this case includes 9 variables but the accuracy as measured by the adjusted *R*^2^ was still very poor: 0.076. The logistic regression model, which was eventually selected as the best model, is discussed in the next section.

#### Machine learning techniques

In addition to the aforementioned regression techniques, we tested also machine learning techniques. In particular, we used the gradient boosting decision tree (GBDT) because it is a supervised model that is particularly good at handing datasets where features span over different scales, as is the case for our survey answers [50, 51]. We implement our GBDT based on the Xgboost algorithm in Python [52]. A randomly selected 20% of the dataset set was used for testing and the rest as a training set. We performed a grid search of the number of estimators using a range of 2*n* with *n* ∈ [1, 10], and set a max depth of the tree in the range of [1, 6]. As shown in the supplementary figure (see S1 Fig), the best achieved accuracy was 66%. We argue that the low results obtained when using machine learning to analyze our data was due to the small number of observations. While this level of accuracy may be acceptable, especially considering the typical needs of large training datasets of machine learning approaches and the small dataset available to us, it is lower than the level of accuracy obtained with the simple logistic regression presented in the next section.

Our conclusion was that all machine learning approaches were inconclusive, arguably due to the small number of observations available for training. Additionally, data visualization techniques, including principal component analysis (PCA) and uniform manifold approximation and projection (UMAP) to reduce dimensionality were applied. However, no distinct clusters were observed (see S2 and S3 Figs in supplementary materials).

#### Logistic regression

For the logistic regression, the value of the risk score, *RI*, had to be converted into a dichotomous variable (*RI*_*b*_) that describes if respondent either does or does not engage in behaviors that leads to risk of Ebola infection. Thus, we first scaled and normalized *RI* with respect to its minimum and maximum values:

$$\begin{array}{c} RI_{n}=\frac{RI-\;\text{min}\;(RI)}{\text{max}\;(RI)-\;\text{min}\;(RI)} \end{array}$$

Second, by using this normalized value of the risk index, *RI*_*n*_ ∈ (0, 1), we set a cutoff value of 0.5 that allowed us to classify individuals in a binary way: individuals that engaged in a risky behavior (*RI*_*n*_ > 0.5, high risk (*RI*_*b*_ = 1)) and individuals that did not engage in a risk behavior (*RI*_*n*_ < 0.5, low risk (*RI*_*b*_ = 0)) from the viewpoint of a possible Ebola infection.

In our logistic regression model, the outcome variable, *Y*, is described as,

$$\begin{array}{c} Y=logit\left(RI_{n}\right)=\text{log}\;(\frac{RI_{n}}{1-RI_{n}})=\beta_{0}+\beta_{1}X_{1}+\beta_{2}X_{2}+\ldots +\beta_{n}X_{n} \end{array}$$ (1)

As shown in the Results section, this regression model provided satisfactory predictive capabilities.

## Results

### Sociodemographic and economic factors underlying the Ebola spillover risk

Following a classification of the spillover risk index into a binary class (high/low risk), we were able to implement a logistic regression (Methods) and investigated both its predictive accuracy and the optimal subset of features to be included. The feature subset was found based on the Akaike Information Criterion (AIC), which estimates the prediction error, therefore the model giving the smallest AIC value was selected [53]. Forward and backward stepwise logistic regression through AIC were applied to select the optimum number of independent variables and to eliminate the variables not contributing significantly to the exposure to risk of spillover.

**Fig 4:**
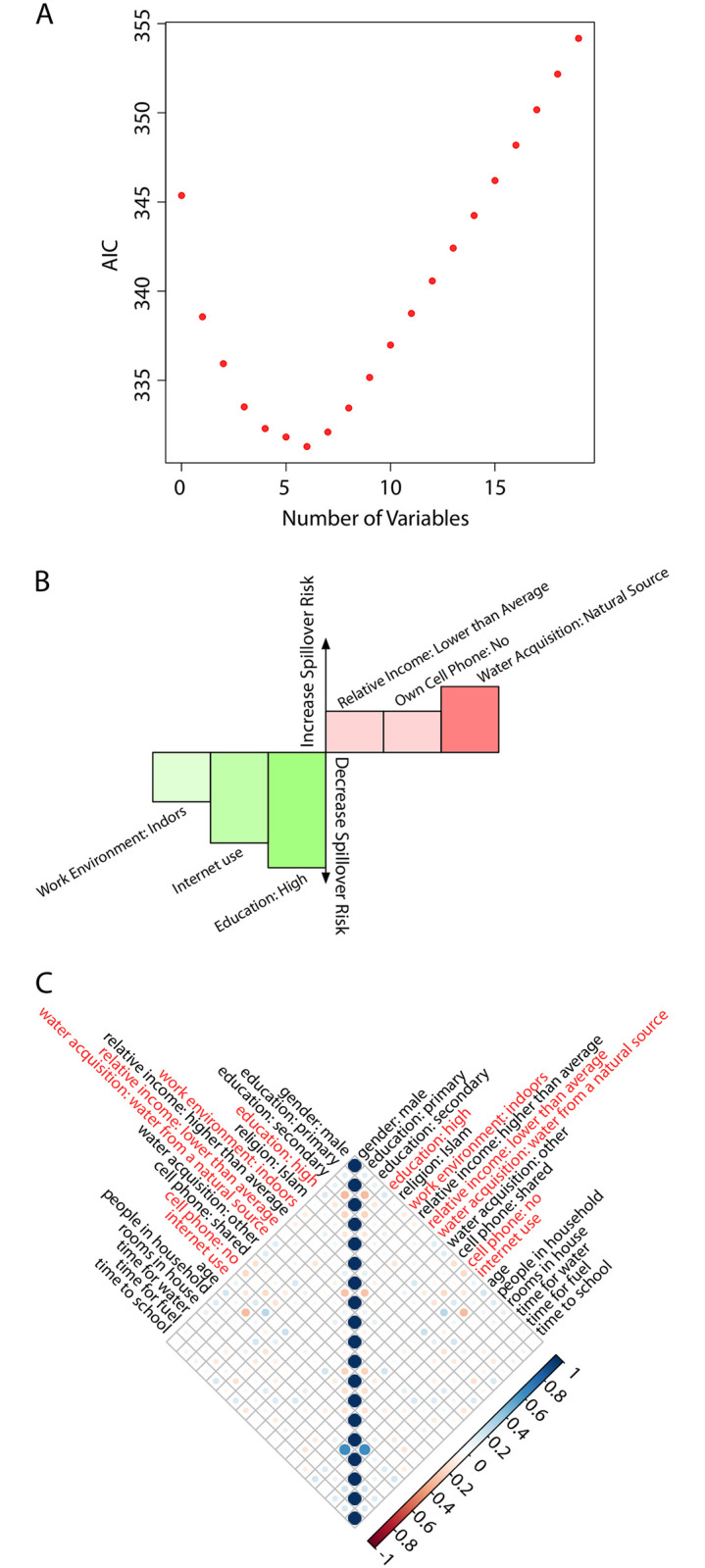
**A: AIC values as a function of the number of variables (features)**. Either starting from a null model and increasing the number of features (forward stepwise logistic regression) or from a complete model and decreasing the number of features (backward stepwise logistic regression), we consistently found that a model with six variables shows a global minimum for AIC (minimum prediction error). **B: Graphical representation of the logistic regression coefficients**. Magnitude of the *β*_*i*_ coefficients (normalized to the maximum) and their sign (positive/negative: red/green). The selected features balance SDE factors that increase or decrease the spillover risk. **C: Graphical representation of the correlation matrix among variables**. Our analysis indicates that there is no significant correlation among variables (red text stand for the selected features in the logistic regression).

Our analyses concluded that a model with six (out of nineteen) features provided a global minimum for the AIC value (Fig 4A and Table 7). Since the adjusted *R*^2^ cannot be used as indicator of the goodness of fit using a logistic regression, we used instead the model accuracy, defined as the percentage of cases where the binary output variable (high/low risk) is correctly predicted by the model. We point out that to measure the model accuracy and robustness we performed a 10-fold cross-validation that was repeated three times with different data partitioning, for a total of 30 analyses using 10% of the data as test samples each time. The accuracy level ranged from 0.5 to 0.81, with an average accuracy of 0.657±0.07. Based on these results, we concluded the model is accurate and robust.

**Table 7:** Selected SDE features with the best predictive capabilities in the logistic regression model.

Feature	*β*_*i*_	*p*-value
education: high	−1.4±0.8	0.07239
work environment: indoors	−0.6±0.4	0.09749
internet use	−1.1±0.8	0.1763
relative income: lower than average	0.5±0.3	0.1185
water acquisition: natural source	0.8±0.4	0.04261
own cell phone: no	0.5±0.3	0.09444

Value of the coefficients *β*_*i*_ for the logistic model shown in Eq (1). ± ranges show the standard error of the corresponding coefficients.

As shown in Table 7 and Fig 4B, SDE factors able to best indicate(or capture) the Ebola spillover risk are features related with education level, work environment, income (including measures of purchasing power), and access to information.

The sign of the coefficient associated with each feature is indicative of the feature being associated with high (positive sign) or low (negative sign) Ebola spillover risk. In that regard, our results revealed that work conditions that decrease possible contact with animals, better educational background, and access to information are factors that decrease the spillover risk. On the other hand, a worse economic status and activities that imply contact with the natural environment increase the chances of infection from a zoonotic source (Fig 4B). To investigate the possible interdependence among predictor variables, we computed their correlation matrix (Fig 4C). No strong correlation between any pairs was found, and the more significant ones are consistent with our expectations (e.g., highest correlation coefficient: 0.63 between “people in household” and “rooms in house”).

**Fig 5:**
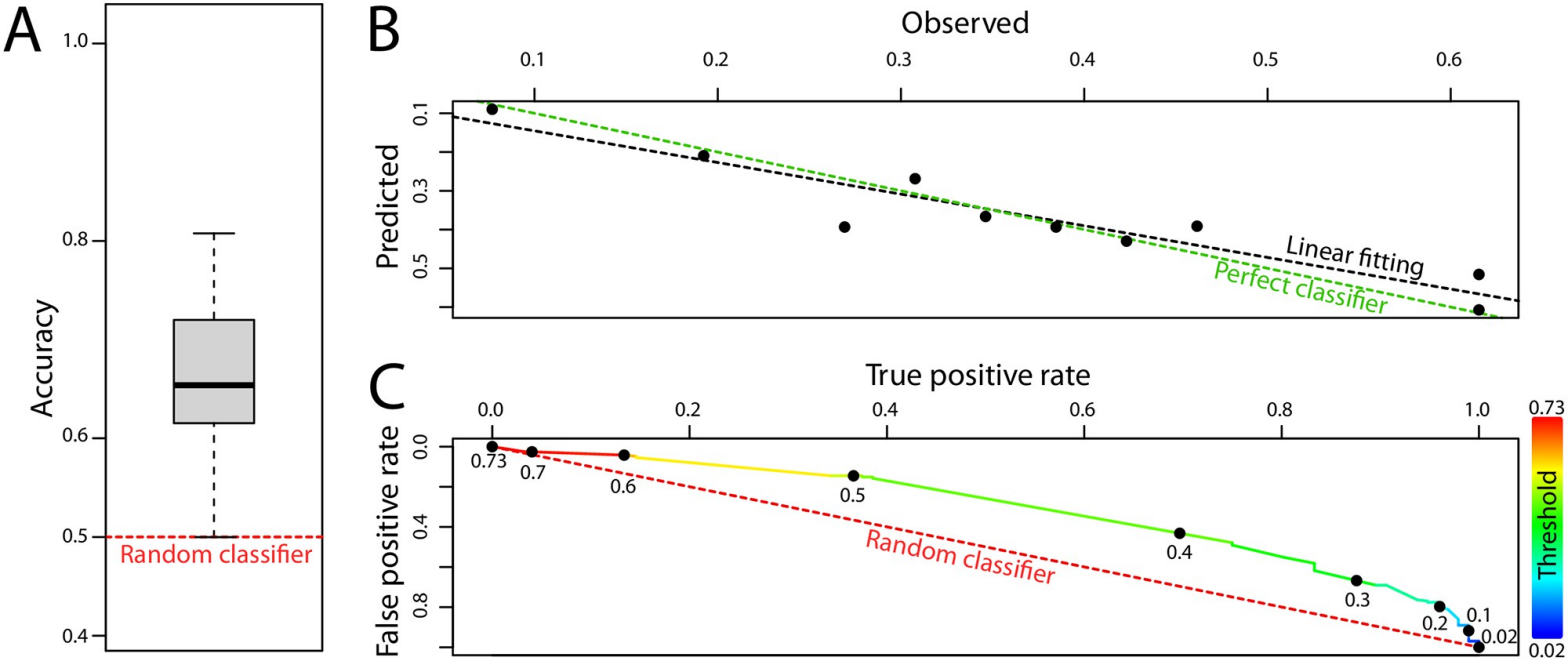
**A: Box plot of the accuracy of the logistic model**. The accuracy, measured as the fraction of correctly predicted spillover risk, is 0.657±0.07. In the plot the wide black line indicates the median. The box delimits the (25%, 75%) percentile interval, and the whiskers represent the minimum and maximum values (no outliers were present in this case). The accuracy analysis was performed repeating a 10-fold cross validation three times (see text). **B: Predicted versus observed spillover risk scores**. The green dotted line is the expected behavior of a perfect classifier and the circles represent the results obtained from our model (see text). The black dotted line is the linear fitting of the points. **C: ROC curve**. As a function of the classifier threshold (color scale) the true versus false positive rate is plotted. The model deviates clearly from a random classifier (red dotted line). Analyses with a threshold larger/smaller than 0.73/0.02 accumulate in top left/bottom right corner of the plot.

We further tested the validity of our logistic model in terms of its predictive capability by different means. To assess the goodness of fit, we used the Hosmer-Lemeshow test [54] that calculates the discrepancy between the predicted and observed risk indexes. The result from the test was not significant (*χ*^2^ = 2.8848) and indicated a satisfactory predictive power (*p* = 0.9414 > 0.05). The successful calibration of predictions was confirmed by analyzing the predicted versus observed risk score (Fig 5B). To that end, we ordered the interviewees by their predicted spillover risk and divided the sorted data into ten equal sets (deciles or bins). For each of these sets we compared the predicted versus observed spillover risk. This analysis confirmed that the regression model is reliable (Fig 5B). Also, given that our model aims at discriminating between the values of a binary outcome (i.e., high risk or low risk), we computed the Receiver Operating Characteristic (ROC) curve [55] in Fig 5C. Our model deviates from a random classifier in a satisfactory way, and the restult of this analysis contributes to justifying the value of the threshold used in the logistic classification (i.e., 0.5). As a way to measure the goodness of the predictive character of our model, we computed the area under the ROC curve (AUC): a perfect classifier would give a value of 1 for this measure and a random classifier a value of 0.5. In our case, we obtained 0.69, which was considered acceptable.

In summary, our logistic regression model is able to identify a reduced set of SDE features to quantify with enough accuracy and in a robust way the Ebola spillover risk in individuals. As shown below, this calibrated model was subsequently used to extrapolate the analysis to the entire country.

### Application of the risk model nation-wide: Infection spillover exposure map

Once our predictive model was properly calibrated and deemed reliable, we aimed at applying it to the entire nation of Sierra Leone. To that end, we used data from the broader survey (SLIHS) conducted by SSL in 2018 and for which responses of individuals are publicly available (∼4 ⋅ 10^4^ interviewees). We designed our survey to include some of the SDE questions in the SLIHS survey. Consequently, we were able to use the SLIHS data set as input in our model and estimate the risk scores of each respondent. Our analysis indicated that the data set was representative of the demographics of rural areas of Sierra Leone (see Methods), which justifies this extrapolation to the rural areas of the country as a whole.

We performed our calculations at the district, *d*, level by computing for each individual, *i*, the spillover risk index using our logistic model: *RI*_*n*_|_*i*,*d*_. By setting a threshold of 0.5 (Methods, see also Fig 5C), the fraction of surveyed individuals at risk of infection in a district reads:

$$\begin{array}{c} p_{d}=\frac{1}{N_{d}}\sum_{i=1}^{N_{d}}\theta (RI_{n}{|}_{i,d}-0.5), \end{array}$$ (2)

where *θ*(⋅) is the Heaviside step function and the sum runs over the *N*_*d*_ individuals that were surveyed in the district. Thus, the density of individuals at risk of being exposed to spillover infection in a district,$\rho_{d}^{I}$, is

$$\begin{array}{c} \rho_{d}^{I}=p_{d}\rho_{d}, \end{array}$$ (3)

*ρ*_*d*_ being the population density in the district [56]. Thus, the infection spillover exposure map is, effectively, the population density map modulated by the spillover risk probability.

**Fig 6:**
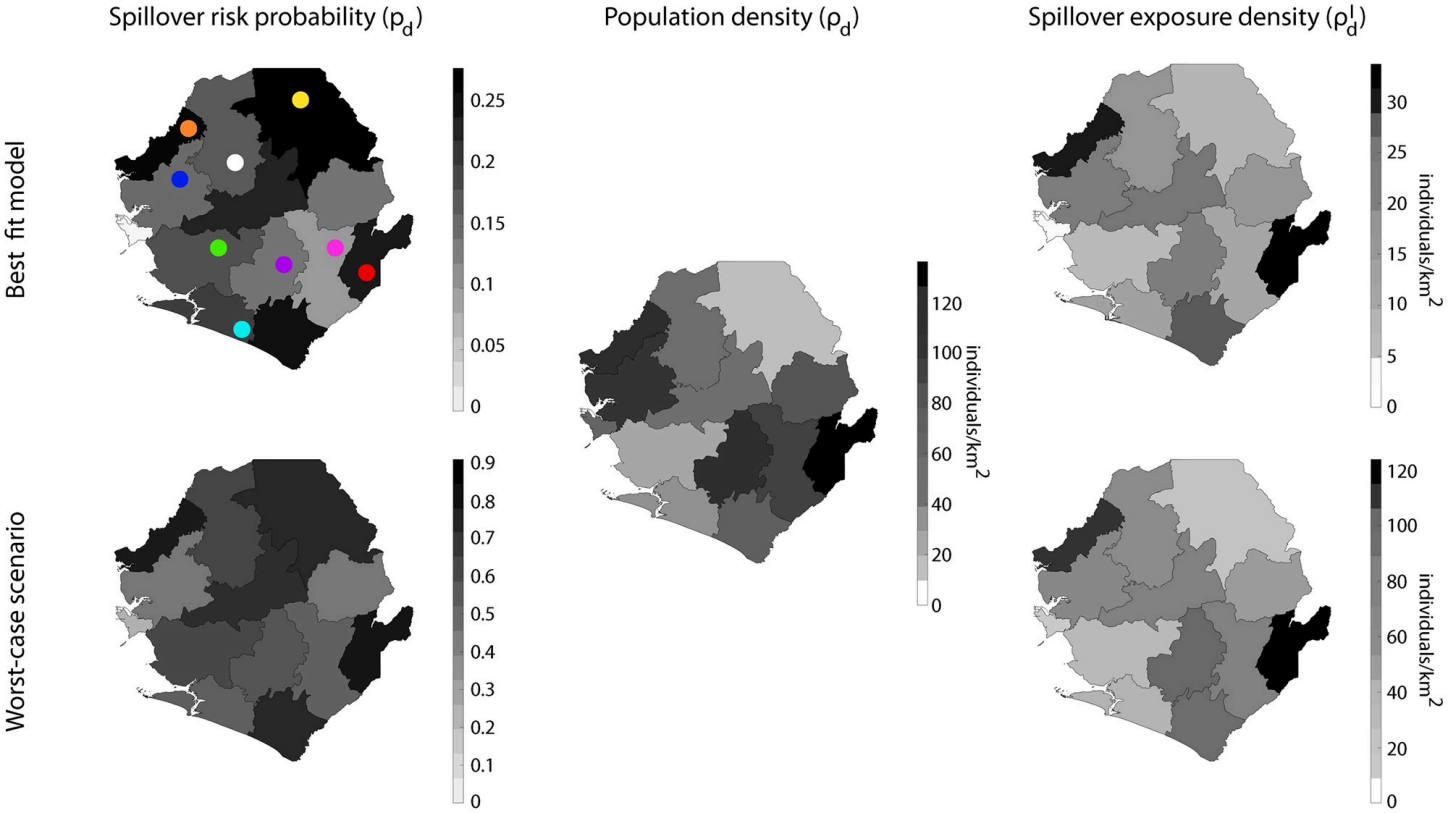
Estimation of the infection spillover map in Sierra Leone by districts. From left to right the figure shows the spillover risk probability (*p*_*d*_), the population density (*ρ*_*d*_), and the infection spillover exposure ($\rho_{d}^{I}$) respectively. In the case of *p*_*d*_ and *ρ*_*d*_ the maps showed on the top stand for the cases of the best fit logistic model and on the bottom the worst-case scenario (see text). District color codes (as shown on top left): Bo (purple), Bombali (white), Bonthe (cyan), Kailahum (red), Kambia (orange), Kenema (pink), Koinadugu (yellow), Moyanba (green), and Port Loko (blue).

Fig 6 shows the infection spillover exposure maps, $\rho_{d}^{I}$, by taking into account the values of *β*_*i*_ in the logistic regression (Table 7) and also the worst-case scenario. To compute the worst-case scenario, we used as model coefficients the values *β*_*i*_ + *ϵ*_*i*_, *ϵ*_*i*_ being the error of the coefficient *β*_*i*_. We point out that the best-case scenario computed by using *β*_*i*_ − *ϵ*_*i*_ predicts no spillover infection, so the associated maps are not included (see Discussion). The maps were created based on publicly available shape files with country profiles and MATLAB scripts were used to generate all figures containing maps [57, 58].

Our data and analyses suggest that Kailahun and Kambia are the rural districts in Sierra Leone with the highest density of individuals exposed to an infection spillover due to SDE factors. This is a combined effect of both high risk spillover probabilities and high population densities. Kailahun is in fact the district where the 2014 Ebola epidemics originated [59]. Koinadugu and Moyamba are two districts with a spillover risk probability that is significantly large. However, their low population density contributes to decrease their spillover exposure. A similar behavior was observed in Bonthe. However, in Port Loko and Bo, the districts the opposite behavior was found: not excessively large risk probabilities combined with high population densities modulate each other and contribute to leave the spillover exposure at average levels. The district of Kenema, which was one of the most severely affected by the 2014 epidemics [60], is not revealed as one of the districts with higher exposure. However, as our model does not account for human-human infective processes and, this result is not particularly surprising. Still, we point out that Kenema neighbors Kailahun, which as mentioned above has one of the largest spillover exposure risks. Arguably, the combined effect of spillover exposure due to zoonotic sources with mobility and human-human infection would have contributed in the past to the large levels of EVD in Kenema. As for the district of Bombali where we ran our survey, average risk probability and population density lead to average spillover risk. Our results also suggest that there is no significant clustering among districts. In order to estimate spatial data clustering, we computed the Moran index (i.e., Moran’s I): a correlation indicator to assess spatial similarity (-1 if there is a perfect dissimilarity, 1 if data are perfectly clustered, and 0 in the case of spatial white noise) [61, 62]. Table 8 shows Moran’s I’s, and the corresponding p-values, for the maps shown in Fig 6. On the one hand, the spillover exposure maps (best fit and worst-case scenario) and the population density map have indexes close to zero (randomness) and data revealed p-values greater than 0.05. On the other hand, the Moran indexes of the spillover risk probability maps indicate a slightly larger degree of clustering that might be significant (p-values smaller than 0.005). This moderate clustering is explainable with demographic trends that go beyond the district boundaries and tune the spillover risk probability.

**Table 8:** Results of Moran’s test.

Map	Moran’s I	p-value
Spillover risk probability (Best fit)	-0.10	0.0025
Spillover risk probability (Worst-case scenario)	0.11	0.00059
Population density	-0.088	0.26
Spillover exposure density (Best fit)	0.034	0.061
Spillover exposure density (Worst-case scenario)	0.033	0.060

Finally, we did not observe significant qualitative changes in the spillover risk probability between the best model and the worst-case scenarios. Nonetheless, we stress the noticeably large levels of spillover risk probability in many districts of the country even in the best model scenario. This points out the necessity, according to our study, of implementing measures that could contribute to lower the spillover risk probability (see Discussion).

## Discussion and conclusions

Herein we have proposed for the first time, to the best of our knowledge, a methodological pipeline to quantify the infection spillover risk probability in individuals and the spillover exposure map at the country level due to SDE factors. Our research contributes to the recent interest in understanding the complexity of epidemic propagation due to confluent effects and for which SDE factors have been proved to be relevant and yet often disregarded. In that regard, previous approaches have focused on evaluating and weighting these factors globally (e.g., at the country level). We instead have focused on the individual level. The advantage of our approach is that it allows scholars and decision makers to obtain a deeper understanding of the social and economic circumstances of individuals to develop a predisposition for risky behaviors in the context of a zoonotic spillover. Thus, our approach can be used to design better targeted campaigns and can help to prioritize resources in space and time (e.g., vaccination, information).

Our results reveal the SDE factors most correlated with the infection spillover probability for individuals (Fig 4). As expected, the educational level, economic level, working conditions, and information access contribute to modulate the risk probability of individuals. Those factors are captured by a reduced number of indicators: work environment, internet use, educational background, relative income, water acquisition source, and cellphone ownership. Our findings showed that gender, religion, and age do not have a major role in modeling the spillover risk probability. Still, some results about these demographic indicators are worth mentioning. Young adults (ages between 18–34) and adults (ages between 34–50) constituted 77% of the investigated sample, but they constitute 86% of the respondents at risk. Also, 50% of the study respondents have an agriculture-related occupation, but when computing the percentage within respondents at risk we obtained 79%. Thus, our model reveals some small biases that suggest that those age ranges and occupations are more susceptible to risky behaviors related to an Ebola infection spillover. Still, we notice that the size of our sample was relatively small and that a larger sample would be required to show that these biases are significant. Related to this last comment, our methodology leverages efforts made regularly in Sierra Leone to measure the demographics. Ideally, in future survey campaigns additional questions to measure risk predisposition could be included by SSL, similar to those included in our local survey. An increased sample size would allow us to refine our results, increase the accuracy, and possibly analyze using by other quantitative methods that were deemed as inaccurate in our study (i.e., machine learning). The findings of the logistic regression model indicate that the only statistically significant variable, using the p-value as a metric, is “water acquisition: natural source” (*p* < 0.05, Table 7). However, there has been an ongoing debate about the possibility of misinterpretation of the p-value with strictly defined thresholds [63, 64]. Some statisticians argue that the interpretation of the p-value is vague and the information coming from p-values of 0.04 and 0.06 is essentially the same [65]. Yet, the former is interpreted to be statistically significant, and the latter is non-significant. The vague classification led us to not consider the *p*-value of 0.05 as a primary criterion for model selection, since our essential aim was to perform as accurately as possible the classification of the spillover risk. As for how representative is our study to capture the spillover risk probability in rural areas at the national level, the evaluation of the reliability of our data revealed that similar trends were obtained in Bombali and the rest of the country. However, some differences were also observed (Fig 3) that might raise questions about the ability to extrapolate our model. This is one of the reasons underlying the exploration of different scenarios (Fig 6). In that regard, our results are qualitatively robust and show a similar relative risk among districts. Nonetheless, we point out that it is certainly possible that if larger surveys are executed in the future, other SDE features could be identified as more representative in terms of their predictive capabilities, following the methodology that we propose. As a possible criticism, the upper and lower bounds of our prediction for the spillover risk probability maps could be considered as too broad: taking as a reference the best fit model, the resulting probability at a given district is approximately four times larger when the worst case scenario is considered. Once more, larger data sets would reduce this variability.

In our study, two different factors are integrated when computing the infection spillover exposure map in Sierra Leone: the spillover risk probability and the population density map. Some districts can actually have a large spillover probability but their low population density helps to diminish their exposure (e.g. Koinadugu). The opposite (relative small spillover probability, and large population density) can lead to similar spillover exposure levels (e.g. Port Loko). Thus, actions should be taken considering the spillover probability as well as the population density of each district. In any case, our model has identified two districts that because of both individual risk and population density are particularly exposed: Kailahun and Kambia. Taking into account that the 2014 epidemics started in Kailahun, more efforts are still needed to lower the spillover exposure there. This study shows the spillover risk probability and spillover risk exposure density without integrating our analysis with the possible spatial relationship among districts. In fact, our analysis revealed lack of clustering among districts. Our results offer simple, easy to interpret and direct conclusions, but in the future, integration of spatial analysis would help to obtain more comprehensive results. In that regard, Bayesian methods are a popular tool to conduct spatial analysis, as they offer a flexible and robust approach, primarily in disease mapping and decision making. [66–69] As a matter of discussion, we stress that our study aims at understanding how SDE factors are related with the Ebola spillover risk. However, a more complete picture of the infection spillover map would require additional drivers (e.g. ecology effects and bat migration habits). In fact, recent studies have established Ebola spillover risk maps in different regions of the African continent where environmental, climatic, and some anthropogenic factors were considered [39]. Still, the authors pointed out that there are still important gaps in the knowledge about the factors leading to infection spillover. We believe that our study accounts for some of those factors and envision that the combination of compartmental models able to provide the density of infected animal host driven by enviroclimatic cues [24] with our approach would lead to a comprehensive assessment of the risk of spillover. In this sense, one of the major contributions of this work is the fact that the complete raw data resulting from our survey campaign in Sierra Leone is provided as additional material to this manuscript, which allows other scholars to perform additional analyses.

Effective allocation of resources is necessary to hinder global epidemics, given the limited health care infrastructure in Sierra Leone and other West African nations. This requires an established priority of what regions are most at risk and therefore most in need of resources. In that regard, our methodology and findings hopefully help to identify the districts which are more susceptible to an infection spillover of Ebola.

## Supporting information

S1 Data Raw data. It collects the data from the surveys. No processing is included in this set. Data on gender, age, location and authors of the interview were considered potentially identifying information by the publisher, so they have been removed from the dataset provided with the article. All the answers of 284 respondents are included.
(XLSX)

S2 Data Cleaned data. First day surveys are excluded. Data on gender, age, location and authors of the interview were considered potentially identifying information by the publisher, so they have been removed from the dataset provided with the article. Data were cleaned without removing any relevant information.
(XLSX)

S3 Data Data with variables included in the analysis. The inputs (SDE variables) and output (Risk Indices) used for the analysis.
(CSV)

S4 Data Data with the variables appearing in the final model. This dataset contains only the variables appearing in the model with the binarized risk indices.
(CSV)

S1 Fig Results of the Xgboost algorithm. (EPS)

S2 Fig Results of the UMAP analysis. (PDF)

S3 Fig Results of the principal component analysis (PCA). (PDF)

S1 File IRB results. Result of Lehigh University’s Institutional Review Board evaluation.
(PDF)

S2 File Consent statement of participants: Informed consent statement that was distributed to all the survey participants, in English and Krio. (PDF)

S3 File Survey instrument: Survey questions and all possible answers, in English and Krio. (PDF)

S4 File PLOS’ questionnaire on inclusivity in global research: A complete copy of PLOS’ questionnaire on inclusivity in global research in our manuscript. (PDF)

S5 File Inclusivity in global research. (DOCX)
