# Supplementary figures and images for: Estimation of Ebola’s spillover infection exposure in Sierra Leone based on sociodemographic and economic factors
Source: PLoS One. 2022 Sep 1;17(9):e0271886. doi: 10.1371/journal.pone.0271886 (PMC9436100; doi:10.1371/journal.pone.0271886)

Plot of Umap Results

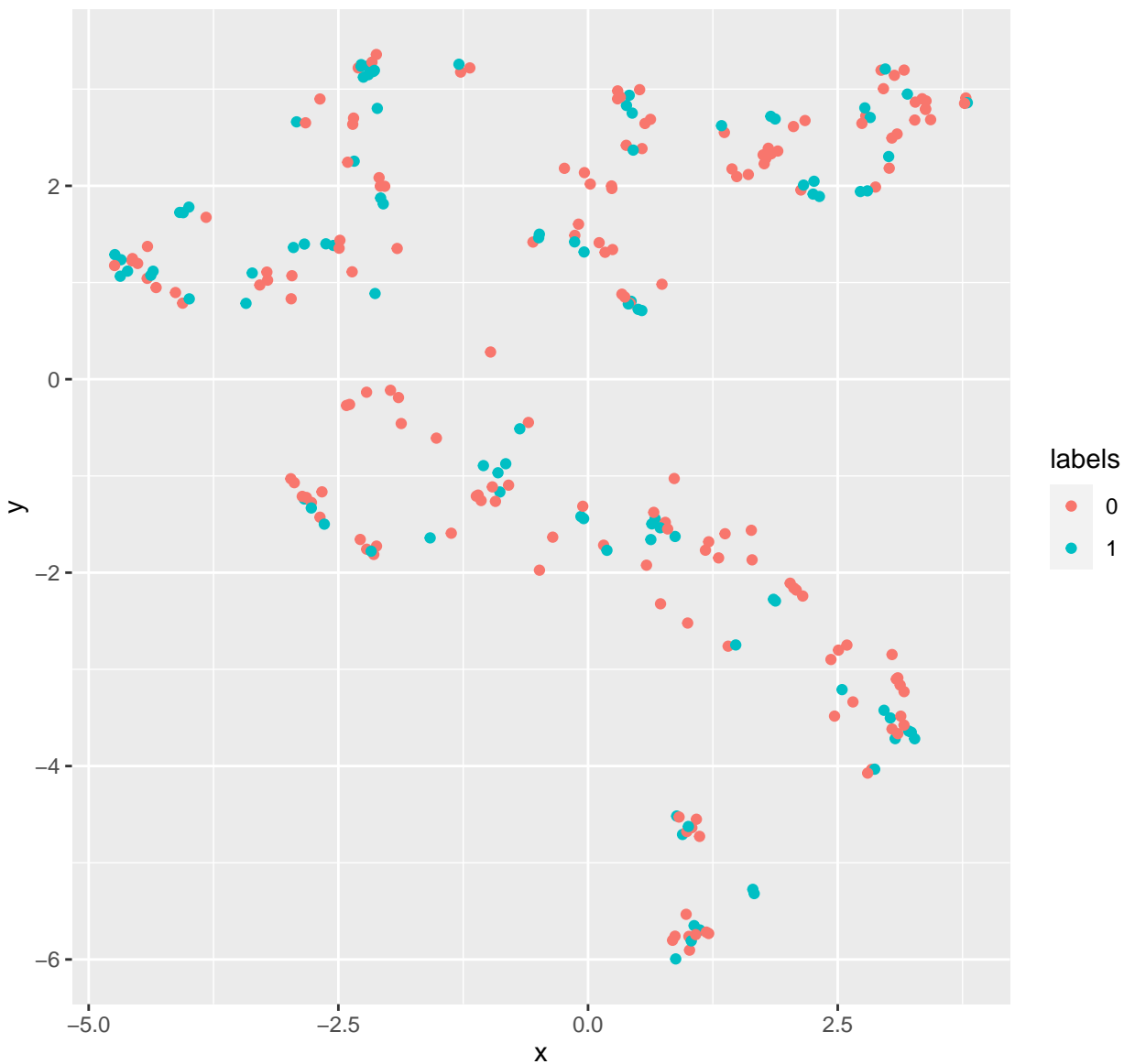

Supplement: S2 Fig — (PDF) [file pone.0271886.s006.pdf]

Plot of PCA Results

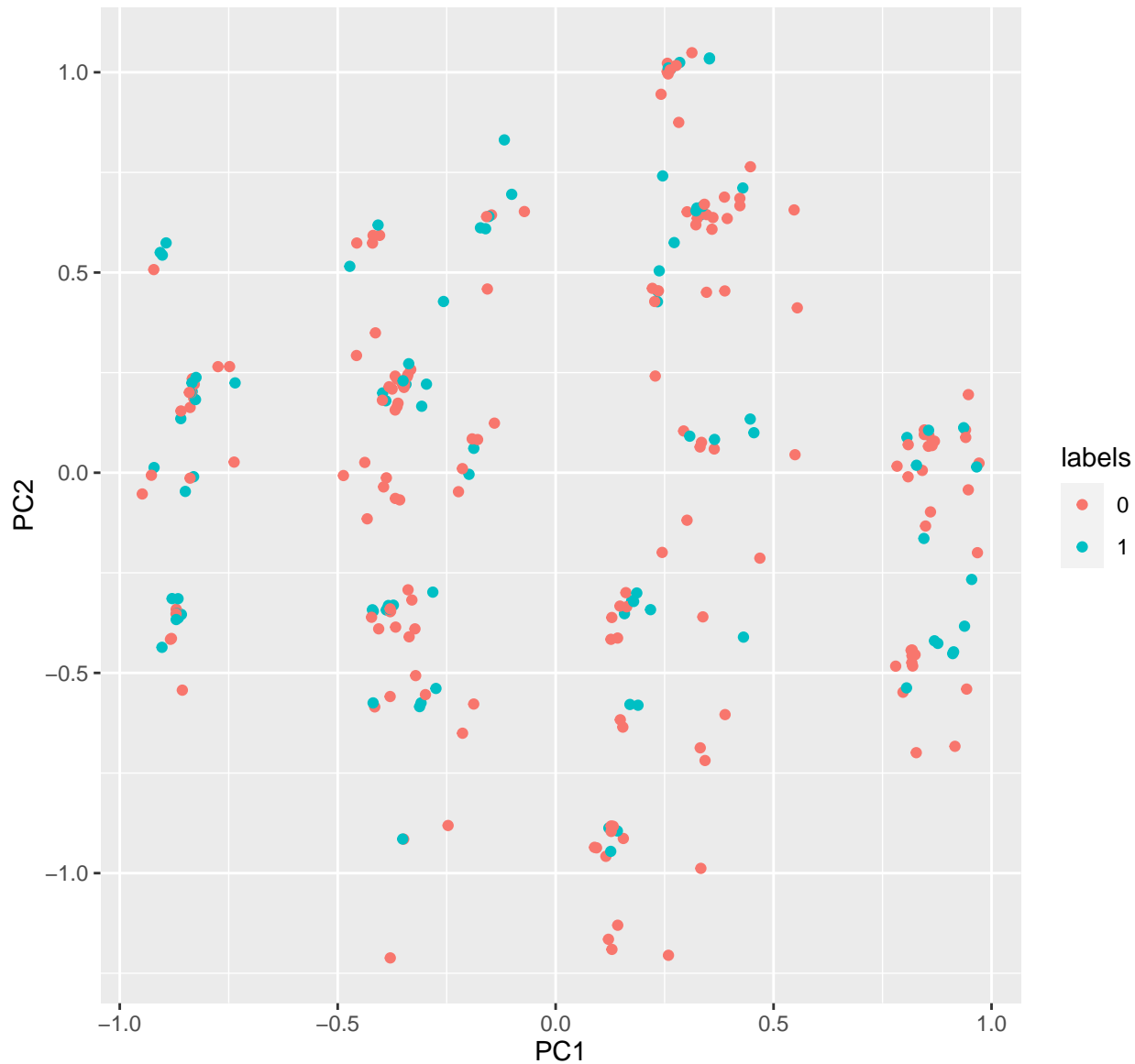

Supplement: S3 Fig — (PDF) [file pone.0271886.s007.pdf]
