# Supplementary material for: Estimation of Ebola’s spillover infection exposure in Sierra Leone based on sociodemographic and economic factors
Source: PLoS One. 2022 Sep 1;17(9):e0271886. doi: 10.1371/journal.pone.0271886 (PMC9436100; doi:10.1371/journal.pone.0271886)
Supplement: S1 File — Result of Lehigh University’s Institutional Review Board evaluation. (PDF) [file pone.0271886.s008.pdf]

Lehigh University logo removed for copyright

Research Integrity
Alumni Memorial Building
27 Memorial Drive West
Bethlehem, PA 18015-3128
(610) 758-2871 Fax (610) 758-5810
https://research.cc.lehigh.edu/research-integrity

DATE:	May 15, 2019
TO:	Paolo Bocchini, PhD
FROM:	Lehigh University Institutional Review Board
STUDY TITLE:	Assessing Socioeconomic Factors Underlying Ebola Infection
IRBNet ID:	1440073-1
SUBMISSION TYPE:	New Project
ACTION:	DETERMINATION OF EXEMPT STATUS
DECISION DATE:	May 15, 2019

Thank you for submitting materials for this research study. The IRB has reviewed the submission and determined that the research is exempt according to federal regulations in the exempt category listed below:

EXEMPT CATEGORY #2:

Research that only includes interactions involving educational tests (cognitive, diagnostic, aptitude, achievement), survey procedures, interview procedures or observation of public behavior (including visual or auditory recording) if at least one of the following criteria is met:

1. *The information obtained is recorded by the investigator in such a manner that the identity of the human subjects cannot be readily ascertained, directly or through identifiers linked to the subjects;*
2. *Any disclosure of the human subjects' responses outside the research would not reasonably place the subjects at risk of criminal or civil liability or be damaging to the subjects' financial standing, employability, educational advancement, or reputation; or*
3. *The information obtained is recorded by the investigator in such a manner that the identity of the human subjects can be readily ascertained, directly or through identifiers linked to the subjects, and the IRB conducted a limited review to determine that there are adequate provisions to protect the privacy of subjects and to maintain the confidentiality of data.*

Note: the only research activities involving children as subjects that may qualify for exemption under categories 2 (i) or (ii) is research involving educational tests or the observation of public behavior when the investigator(s) do not participate. Exempt category 2 (iii) may not be applied to research with children.

Study Changes or Amendments: Any changes or amendments to the approved study must be submitted to the IRB via an Amendment/Modification submission in IRBNet. Proposed changes may not be initiated without IRB approval, except when necessary to eliminate immediate hazards to subjects.

Generated on IRBNet

Reporting Unanticipated Problems: All unanticipated problems involving risks to subjects or others must be reported to the IRB within five business days via a Reportable New Information submission in IRBNet. All events that are considered reportable to the IRB, including noncompliance, subject complaints, and subject injury, are listed in the Lehigh University IRB's Policy: Reporting Unanticipated Problems/Adverse Events to the IRB. It is the investigator's responsibility to be aware of and follow any additional sponsor reporting requirements.

Please direct questions about this approval to Erin Karahuta, Research Integrity Specialist, at 610-758-2199 or inirb@lehigh.edu. Please include the study title and IRBNet ID in all correspondence.

Generated on IRBNet
