# Supplementary material for: Estimation of Ebola’s spillover infection exposure in Sierra Leone based on sociodemographic and economic factors
Source: PLoS One. 2022 Sep 1;17(9):e0271886. doi: 10.1371/journal.pone.0271886 (PMC9436100; doi:10.1371/journal.pone.0271886)
Supplement: S2 File — (PDF) [file pone.0271886.s009.pdf]

Consent Statement – English

Lehigh University logo removed for copyright

Hello. I am working with Lehigh University to conduct a study on the risk factors for transmission of the Ebola virus in order to prevent and combat future outbreaks. We will ask you some questions on yourself and your daily habits. The information we collect will help us and other civil society organizations better understand how to effectively prevent the transmission of the disease and allocate resources to areas at risk. The interview will take about 30 minutes. All of the information you share with us will be kept confidential – meaning that we will not link your name in any reports or identify you as the respondent in any other way. Your anonymous data may be shared with other researchers for future research. Your participation in this study is voluntary, and you will not be paid. You may choose to stop the interview at any point or refuse to answer questions you do not feel comfortable responding to. There are no right or wrong responses. Therefore we encourage you to be honest and truthful in your responses so that we can accurately understand the on-the-ground situation.

You may contact the Principal Investigators for further questions or to report issues at:
+16107583066 or pab409@lehigh.edu (Dr. Paolo Bocchini) or
+16107582878 or jab614@lehigh.edu (Dr. Javier Buceta).

You may report problems that may result from your participation or direct questions in regard to your rights as a subject in this study to Lehigh University's Office of Research Integrity at +16107582871 or inirb@lehigh.edu. All reports or correspondence will be kept confidential.

U willingness word – Krio

Lehigh University logo removed for copyright

Hello, I da work for Lehigh University for make a study on the risk thing for transfer of sick (disease) for any other sickness way e go cam. We dae ask you how u dae due u dae to dae habits. De information way we dae collect go help we and other people way dem dae tok for we for bette understand how dem go control de sickness en pass one to other person en for put money na area way na treat for well body.de question way we dae ask go last for 30 minutes.All the answer way una go give we go keep pan secrets. Way e mean we nor give u name na any reports or as somebody way give we the answers. De record way we take from we give other people or organization for due more findings. U attention na dis findings nor go give u any money. So any time u want stop for give we information u ken stop if u nor feel fine. No correct or wrong answer nor dae we want all. We go want beg u for le e go be honest and tell D true na u answer way u dae give correct so dat we go know watin dae na d ground.

If u want for know more u go contact the Big one dem for other questions dem or for report things dem.
+16107583066 or pab409@lehigh.edu (Dr. Paolo Bocchini) or
+16107582878 or jab614@lehigh.edu (Dr. Javier Buceta).

U kin complain anything way e go cam because u take part or u direct involvement as person way e take parts because na dis study for give information to Lehigh University`s office of Research Integrity at for find out +16107582871 or inirb@lehigh.edu. All u report or answers go be keep secret.

Translation of the oral questions to Krio

Hello, I da work for Lehigh University for make a study on the risk thing for transfer of sick (disease) for any other sickness way e go cam. We dae ask you how u dae due u dae to dae habits. De information way we dae collect go help we and other people way dem dae tok for we for bette understand how dem go control de sickness en pass one to other person en for put money na area way na treat for well body.de question way we dae ask go last for 30 minutes.All the answer way una go give we go keep pan secrets. Way e mean we nor give u name na any reports or as somebody way give we the answers. De record way we take from we give other people or organization for due more findings. U attention na dis findings nor go give u any money. So any time u want stop for give we information u ken stop if u nor feel fine. No correct or wrong answer nor dae we want all. We go want beg u for le e go be honest and tell D true na u answer way u dae give correct so dat we go know watin dae na d ground.
If u want for know more u go contact the Big one dem for other questions dem or for report things dem.
+16107583066 or pab409@lehigh.edu (Dr. Paolo Bocchini) or
+16107582878 or jab614@lehigh.edu (Dr. Javier Buceta).
U kin complain anything way e go cam because u take part or u direct involvement as person way e take parts because na dis study for give information to Lehigh University`s office of Research Integrity at for find out +16107582871 or inirb@lehigh.edu. All u report or answers go be keep secret.

Do you have any questions? > u get any question for ask

Do I have permission to continue with the interview? > er kin go ahead for ask u

A.

1. **In what district were you born? < whose district u born**
2. **What is your gender? < watin u na sex**
3. **How old are you?< watin na u age**
4. **What type of education did you receive? (Read Options) < whose kind learning u get**
    a. No formal education < u nor go school
    b. Some primary school < na primary school
    c. Completed primary school < u done primary school
    d. Completed Junior Secondary School (JSS) < u done junior secondary school
    e. Completed Senior Secondary School (SSS) < u done senior secondary school
    f. Completed Diploma or Postsecondary Training < u done diploma or na sckills work u due.
    g. Completed Bachelors < u done due first Degree
    h. Completed Masters or Doctorate < u done due u masters or Doctorate Degree
    i. Other:________________________________ < u get any other paper

j. Declined to answer < u want for stop answer dis question

5. **What is your religion? < watin u religion**
6. **What is your occupation? < whose work u dae due**
7. **Do you work indoors or outdoors? <na in side u dae work or out side**

7b. If Outdoors: Are there wild animals around you when you work, such as rabbits, bats, monkeys…?< if na outside u dae see wild animals dem around d side way u dae work lek bush arata,bats dem,monkey dem

8. **Is your household income lower / equal / higher than average? Waitin una dae eat e bette or e nor bette or e bock or e due for una**

<u>B.</u>

1. **How many members are in the household? < how many people den dae na u house**
2. **How many rooms are in the household? < how many room den dae na d house**
3. **How many Family units live in your house? < na una so so dae na d house or na different people**
4. **How do you get water for your household? < how una dae water for d house**
5. **How long does it take to get water for your house in the dry season?< how many minutes e dae take una for get water during dry season**
6. **How long does it take to get cooking fuel to your house? < how many minutes e take for find wood or coal for cook na house**
7. **How often do you clean the surfaces from which you cook or eat? < how many time u d clean side way u dae cook or eats**
8. **How often do you use bleach/chemicals to clean your house? < how many time u dae use chemical way dae clean d house**
9. **How often do you go to Makeni?<how many time u kin go makeni**
10. **How far do you live from the nearest highway? < how far u dae live from d may road**
11. **How far do children in the house travel to primary school? < how long den dae waker for go na primary school**
12. **How often do you go to the market in Makeni? < how many time u dae go na big market in side makeni?**

13. How often do you use the internet? < how many time u dae use internet

14. Do you own a cell phone? < u get phone for u self

C.

1. How often do you spend time in wooded areas/forests/jungles? < how long u spend time na bush

2. Do you know of any caves? <u know whose cave dey

2b. IF YES: How often do you go to caves?<if na true how many time u go na cave

3. How often do you wash with soap?<how many time u dae wash with soap

4. How many meals do you eat in a typical day? < how many time u dae eat for d day

5. When you eat fruit, do you check if it has been bitten by animals?<way u dae eat fruit u dae watch if animals den dem bet am

6. Which one of these proteins do you eat twice or more a week? (Check all that apply) (Read options)<whose one pan dese protein den way u dae eats two times or more for d week

- ☐ Beans
- ☐ Fish
- ☐ Chicken
- ☐ Beef
- ☐ Bushmeat (Bats, Antelope, Monkey, Anteater, Rodents, etc.)
- ☐ Other: ________________________________<any other one dae way we nor callam
- ☐ None<natin pan d one dem way we call
- ☐ Declined to Answer<le we lef dis question

7. What is your favorite form of meat? < whose beaf u lek

8. Do you see bushmeat sellers in your village? (Bats, Antelope, Monkey, Anteater, Rodents, etc.) < u dae see person way dae sell bush beaf

9. How often do you eat Bushmeat? (Bats, Antelope, Monkey, Anteater, Rodents, etc.) < how many time u dae eat bush beaf

10. What is your favorite form of Bushmeat? < whose bush beat way u lek

11. Do you clean your hands before eating? < u kin wash u hand befo u eat

12. **How often do you have contacts with sick people?<how many times way u dae touch sick person**
13. **How often do you spend time in places where bats nest? < how long u kin spend na place way bat den house**
14. **How often do you have contact with someone else's blood or bodily fluids?**
15. **Do you believe that touching raw meat or any live animal could spread disease? < u belief say if u touch raw meat or any live animals go scatter sick**
16. **Do you believe that eating bushmeat could spread disease? < u belief say way u eat bushmeat go scatter sickness**

<u>D.</u>

1. **What is the best way to characterize the environment surrounding your village? (Check all that apply) (Read options) < how u go able tell we how u village in setting dey**
   - ☐ Wooded/Forest area < na bush or forest
   - ☐ Jungle < na side way bad animal den dey
   - ☐ Urban environment < na big town
   - ☐ Barren land < na reserved bush
   - ☐ Other < any other thing
   - ☐ Declined to answer<le we leef dis answer
2. **Are there bats around your house? < bats den dae near u house**
3. **Has the number of bats always been the same? < d bat number na d same**

3b. When did the number of bats changed? (Skip if Yes to Question 3) < whose time d bats dem number add or reduce

4. **Has there been any deforestation conducted around where you live? < den done dae cut bock sticks dem down na d area way u dae**

<u>E.</u>

1. **What are the ways in which a person get Ebola? (Check all that apply) (Open Question) <watin na d ways dem way person dae get Ebola**
2. **Do you think a person could get Ebola from an animal? < u think say person dae get Ebola from animal dem**

2b. IF YES: How could a person get Ebola from an animal? (Check all that apply) (Read options) < if na true how person dae get Ebola from animal

- ☐ Having an animal as a pet < for meand animal dem
- ☐ Eating any meat<for eat any beaf or fowl
- ☐ Eating bushmeat < for go dae eat bushbeaf
- ☐ Watching an animal<for dae look animal
- ☐ Eating fruits bitten by an animal<for go dae eat den fruit way animal done bet lef
- ☐ Hunting<for go dae go hunting
- ☐ Preparing bushmeat as a meal < for go dae make bushbeaf as food
- ☐ Other:________________ < any other one

3. **In general, how do you think a person avoids Ebola? (Check all that apply) (Read Options)<na u sense how u think person go away from Ebola**

- ☐ Brushing their teeth<for go dae brush u teeth
- ☐ Sleeping under a mosquito net<for go dae sleep under mosquito tent
- ☐ Avoiding contact with blood and bodily fluids < nor for touch blood and any water way dae comute from d body
- ☐ Drinking tea<for go dae drink tea
- ☐ Staying inside when it rains<for dae inside d time way rain dae cam
- ☐ Not touching anyone with the disease < nor touch person way sick
- ☐ Clean themselves with soap and water < wash with soap and water
- ☐ Avoiding funerals or burial rituals < nor go pan burying or sara
- ☐ Drinking only tap water<for go dae drink water well water
- ☐ Avoiding the forest/woods < nor go na bush
- ☐ Other: ______________________________ < any other thing
- ☐ I don't know < er nor no
- ☐ Declined to answer < le we lef d answer

4. **What actions do you take to avoid Ebola when you know there is an outbreak? (Check all that apply) (Read Options)<watin u go due for make u nor get Ebola way u know say Ebola dae**

- ☐ Brushing your teeth<for go dae brush u teeth
- ☐ Sleeping under a mosquito net< for go dae sleep under mosquito tent

- ☐ Avoiding contact with blood and bodily fluids < nor for touch blood and any water way dae comute from d body
- ☐ Drinking tea< for go dae drink tea
- ☐ Staying inside when it rains < for dae inside d time way rain dae cam
- ☐ Not touching anyone with the disease < nor touch person way sick
- ☐ Clean yourself with soap and water < wash with soap and water
- ☐ Avoiding funerals or burial rituals < nor go pan burying or sara
- ☐ Drinking only tap water < for go dae drink water well water
- ☐ Avoiding the forest/woods < nor go na bush
- ☐ Other: ________________________________ < any other thing
- ☐ I don’t know < er nor no
- ☐ Declined to answer < le we lef d answer

5. Have you ever had Ebola? < u ever get Ebola

6. Do you have a relative or friend who had Ebola? < u get any family or padi way e ben done get Ebola

6b. (Follow up: If yes to question # 7) Who had the disease < if na true na who dat

7. **Do you believe that you can get Ebola from bushmeat? < u belief say u go get Ebola from bushbeaf**
